# Prediction Consistency Regularization for Learning with Noise Labels Based on Contrastive Clustering

**DOI:** 10.3390/e26040308

**Published:** 2024-03-30

**Authors:** Xinkai Sun, Sanguo Zhang, Shuangge Ma

**Affiliations:** 1 School of Mathematics Sciences, University of Chinese Academy of Sciences, Beijing 100049, China; sunxinkai18@mails.ucas.ac.cn (X.S.); sgzhang@ucas.ac.cn (S.Z.); 2 Key Laboratory of Big Data Mining and Knowledge Management, Chinese Academy of Sciences, Beijing 100049, China; 3 Department of Biostatistics, Yale School of Public Health, New Haven, CT 06510, USA

**Keywords:** deep learning, noisy label, consistency regularization, contrastive learning

## Abstract

In the classification task, label noise has a significant impact on models’ performance, primarily manifested in the disruption of prediction consistency, thereby reducing the classification accuracy. This work introduces a novel prediction consistency regularization that mitigates the impact of label noise on neural networks by imposing constraints on the prediction consistency of similar samples. However, determining which samples should be similar is a primary challenge. We formalize the similar sample identification as a clustering problem and employ twin contrastive clustering (TCC) to address this issue. To ensure similarity between samples within each cluster, we enhance TCC by adjusting clustering prior to distribution using label information. Based on the adjusted TCC’s clustering results, we first construct the prototype for each cluster and then formulate a prototype-based regularization term to enhance prediction consistency for the prototype within each cluster and counteract the adverse effects of label noise. We conducted comprehensive experiments using benchmark datasets to evaluate the effectiveness of our method under various scenarios with different noise rates. The results explicitly demonstrate the enhancement in classification accuracy. Subsequent analytical experiments confirm that the proposed regularization term effectively mitigates noise and that the adjusted TCC enhances the quality of similar sample recognition.

## 1. Introduction

In recent years, neural network-based methods have achieved unprecedented success in the fundamental machine-learning task of classification [1,2,3]. However, the effectiveness of these models depends on the quality of labeled datasets, which often contain mistakes known as label noise, resulting from various factors [4]. For example, automatically collecting image labels through methods like web scraping cannot guarantee the correctness of all labels [5]. Similarly, in biostatistics, measurement errors are quite common [6]. The capable parameters of neural networks grant them significant model capacity, but they also make it easy for the networks to overfit noisy labels, ultimately resulting in poor model performance. Developing methods suitable for learning with noisy labels has significant implications for fields such as image analysis and medical applications [4].

A well-trained model is expected to yield consistent outputs for similar inputs. However, recent work [7] reveals that models trained on datasets with label noise exhibit significant variations in predictions when faced with two different augmentations of the same image. In classification tasks, the consistency between probability distributions can be measured using the cross-entropy function. From this perspective, label noise leads to an abnormally increased cross-entropy between the predicted probability distributions for similar inputs. To address this anomaly, recent research [7,8] suggests introducing regularization terms on top of the classification loss to combat the adverse effects of noise. These regularization terms, known as prediction consistency regularization terms, aim to minimize prediction variance among similar samples. However, building consistency through regularization relies on identifying samples in the training dataset that share similar labels. The mismatch between assigned labels and their true counterparts hinders the accurate identification of all samples sharing the same true label. A more general alternative is to consider samples that are close enough as samples sharing the same labels, effectively transforming the problem from identifying samples with the same label to recognizing similar samples.

Determining sample similarity in datasets with straightforward structures is relatively easy. However, for more complex data types, such as images, this task becomes more challenging. In the case of such complex-structure data, a feasible approach is to map the data into a representation space with a simpler structure and then search for similar samples by analyzing the relationships between these representations. Recently, contrastive learning [9,10,11,12] has gained significant attention as a set of representation learning methods. It can provide representations that are independent of label noise and have the potential to identify similar samples. Nevertheless, contrastive learning is primarily used for unsupervised pretraining, with its core objective being the acquisition of transferable representations. This objective differs significantly from the core goal of classification tasks and brings two potential risks when applying self-supervised learning to label-noise classification: (1). The process of self-supervised representation learning does not involve label information, implying that samples with similar self-supervised representations may not necessarily share the same labels. (2). Mainstream contrastive learning frameworks emphasize obtaining transferable representations; then, identifying similar samples requires computing similarities between representations of all samples, leading to additional computational burdens.

This work proposes the twin-contrastive-clustering-based prediction consistency regularization (TPCR) to effectively handle label noise for image data. The proposed method consists of two main components. On the one hand, to accurately and efficiently identify similar samples and reduce potential risks associated with self-supervised learning, TPCR adopts twin contrastive clustering (TCC) [12] as the framework for representation learning. We improve TCC by integrating valuable information, enabling it to produce representations that reflect label consistency, thereby addressing the first potential risk. Since TCC’s pretext task involves clustering input samples into different groups, samples belonging to the same cluster can be considered inherently similar without the need for additional calculations, thus avoiding the second potential risk. On the other hand, based on the refined TCC’s clustering results, this paper designs a prototype-based regularization method that improves classification consistency within the same cluster by penalizing the cross-entropy between model outputs and the prototypes. Ultimately, these measures help alleviate the adverse effects of label noise on model performance, leading to improved model performance.

The main structure of this paper includes the following sections: In Section 2, Section 2.1 discusses related work on noisy label classification, while Section 2.2 introduces contrastive learning. In Section 3, Section 3.1 introduces the relevant notation; Section 3.2 describes TCC; Section 3.3 presents the adaptive modifications made to TCC; and Section 3.4 presents the proposed regularization term and provides an overview of the overall model training process. Section 4 focuses on the experiments, with Section 4.1 discussing the performance of the proposed method under simulated noise, Section 4.2 presenting the performance on real noisy data, Section 4.3 analyzing the sensitivity to key hyperparameters, and Section 4.4 conducting ablation experiments on the proposed components. Finally, we summarize the paper and discuss future research directions.

## 2. Related Works

### 2.1. Learning with Noisy Labels

We focus on methodologies pertaining to noise-robust loss functions, which align closely with the framework of the proposed method. Ghosh et al. [13] proved that mean-absolute error (MAE)-based loss functions are tolerant to label noise under specific conditions, while traditional cross-entropy loss exhibits high susceptibility to label noise. The innovative mean-absolute error (IMAE) [14] introduced nonlinear transformations into MAE’s weighting scheme through the exponential function, establishing a more effective learning process for extracting meaningful patterns. Expanding the scope of noise-tolerant loss functions, Liu et al. [15] generalize the robustness of existing binary loss functions to accommodate multi-category classification scenarios. Furthermore, the generalized cross-entropy (GCE) [16] introduces a unique perspective by employing the negative Box-Cox transformation as a loss function. The symmetric cross-entropy (SCE) [17] introduces a novel component in the form of reverse cross-entropy, enhancing the conventional loss by promoting symmetry in predictions. The generalized Jensen-Shannon divergence (GJS) [7] is applied to improve sample-level prediction consistency. The neighborhood consistency regularization (NCR) [8] introduces a regularization term aimed at reducing the difference between the prediction of each sample and those of their nearest neighbors. Another innovative approach is embodied by early learning regularization (ELR) [18]. ELR introduces a distinctive regularization term that guides the model towards reproducing its past outputs, on the early-learning phenomenon [19].

While MAE [13], IMAE [14], GCE [16], and SCE [17] mainly focus on modifying the cross-entropy function and may struggle in extreme label noise conditions, GJS [7], NCR [8] and ELR [18], emphasize prediction consistency, with GJS and ELR concentrating on individual-sample-level consistency and NCR on nearest neighbors, which may not suffice in severe noise scenarios. TPCR uniquely targets cluster-level prediction consistency, setting it apart from the aforementioned approaches.

Additionally, Decoupling [20], Co-teaching [21], Co-teaching+ [22], JoCoR [23], NCT [24] and Co-learning [25] rely on the integration of multiple models or tasks that are similar to TPCR. In contrast, TPCR employs clustering as its complementary task, illustrating a notable distinction from these methodologies.

### 2.2. Contrastive Learning

Contrastive learning is an unsupervised learning method with the goal of pre-training representations that can be fine-tuned for downstream tasks [9]. Pretext tasks in existing contrastive learning methods can be broadly categorized into contrastive-instance and clustering-based [10]. Specifically, methods like MoCo [9], SimCLR [11], BoyL [26], and SimSiam [27] fall under the contrastive-instance category, while Swav [10], DeepCluster [28], PCL [29], and TCC [12] belong to the clustering-based approach.

In recent years, a notable trend has emerged in the form of contrastive-learning-based methodologies tailored to address the challenges posed by noisy labels. These innovative methods, including C2D [30] and the method in [31], harness the power of contrastive learning for pre-trained model initialization. Furthermore, MOIT [32], SelCL [33], Mopro [34], ProtoMix [35], and TCL [36] exploit representations derived from contrastive learning to selectively identify confident samples or generate pseudo-labels. Ctrr [37] introduces a novel contrastive regularization mechanism applied to representations. Finally, the co-learning method [25] represents a fusion of label-dependent information from supervised learning with feature-dependent insights derived from contrastive learning, thereby amalgamating the strengths of both paradigms.

These methods are based on the contrastive-instance framework, and the clustering-based framework has not been fully utilized; moreover, the above methods are highly dependent on calculating similarity between samples, which introduces an additional computational overhead. In contrast to these methods, TPCR utilizes a clustering-based framework and requires no additional computation.

## 3. Method

In this section, we detail our proposed methodology, beginning with an overview of the noise classification problem and notations in Section 3.1, followed by an explanation of twin contrastive clustering (TCC) [12] in Section 3.2. These sections serve as an introduction to the foundation for TPCR. Section 3.3 presents modifications to TCC informed by label information, while Section 3.4 presents the novel regularization terms based on the clustering outcomes of the adjusted TCC.

### 3.1. Problem Formulation

Considering a classification problem with *C* classes, denote the input space as $X\subset R^{d_{1}}$ and the label space as Y={1,2,⋯,C}. Generally, models are trained on the clean dataset denoted as $D=\{\left(x_{1},y_{1}\right),\left(x_{2},y_{2}\right),\cdots ,\left(x_{N},y_{N}\right)\}$, with $x_{i}\in X$, $y_{i}\in Y$ and *N* representing the dataset’s sample size. When learning with noisy labels, we only have access to the noisy dataset $\tilde{D}=\left\{\left(x_{1},{\tilde{y}}_{1}\right),\left(x_{2},{\tilde{y}}_{2}\right),\cdots ,\left(x_{N},{\tilde{y}}_{N}\right)\right\}$, where ${\tilde{y}}_{i}\in Y$ is noisy; that is, some of ${\tilde{y}}_{i}\neq y_{i}$ and do not correctly reflect the visual content of the corresponding input. During training, only noisy labels are available, and it remains unknown whether $\tilde{y_{i}}$ is noisy ( $\tilde{y_{i}}\neq y_{i})$ or clean ($\tilde{y_{i}}=y_{i}$). The objective is to train a model that achieves high accuracy on the true labels despite the presence of an unspecified number of noisy labels in the training set.

The neural network model for this classification task is denoted as $f_{\theta }:X\to R^{C}$, where θ is the trainable parameters of the network. This model captures the conditional probability distribution of $y_{i}$. Specifically, the model first maps the input $x_{i}$ to a logits vector $w_{i}=f_{\theta }\left(x_{i}\right)\in R^{C}$. Subsequently, a softmax operation is applied to transform $w_{i}$ into ${\hat{y}}_{i}={\left({\hat{y}}_{i1},{\hat{y}}_{i2},\cdots ,{\hat{y}}_{iC}\right)}^{⊤}\in R^{C}$, where ${\hat{y}}_{ic}\left(1\leq c\leq C\right)$ can be viewed as the probability of $x_{i}$ belonging to *c*-th category. When learning with noisy labels, this model employs a noisy classification loss function:

(1) $$L_{ce}=\frac{1}{N}\sum_{i=1}^{N}\ell_{ce}\left({\tilde{y}}_{i},{\hat{y}}_{i}\right)=-\frac{1}{N}\sum_{i=1}^{N}{\tilde{y}}_{i}^{⊤}\log\left({\hat{y}}_{i}\right)\;,$$

where $\ell_{ce}$ is the cross-entropy function and ${\tilde{y}}_{i}$ is the one-hot vector corresponding to ${\tilde{y}}_{i}$. Notably, ${\tilde{y}}_{i}$ and ${\hat{y}}_{i}$ can also represent the probability mass function of the categorical distribution. For the sake of brevity, we will use the ‘probability vector’ to refer to the probability mass function of a categorical distribution in the subsequent sections. With label noise, optimization of Equation (1) leads to overfitting label noise, which reduces the prediction accuracy on clean labels.

### 3.2. Twin Contrastive Clustering

In order to identify similar samples, we need to obtain the representation of samples and conduct clustering. This study adopts twin contrastive clustering (TCC) [12] as a contrastive learning framework. Prior to introducing TCC, we first describe the contrastive-instance method that underpins TCC’s methodology.

Contrastive learning leverages the unlabeled dataset $D_{x}=\left\{x_{1},x_{2},\cdots ,x_{N}\right\}$, obtained by ignoring label information from datasets D or $\tilde{D}$. Contrastive learning relies on pretext tasks for supervision [38], broadly categorized into contrastive-instance and clustering-based categories. The contrastive-instance approach involves identifying two augmented versions of the same input as belonging to the same category, serving as single-sample recognition. Specifically, after random augmentations, $x_{i}$ yields two variants: $x_{i}^{\left(1\right)}$ and $x_{i}^{\left(2\right)}$, which are then transformed by a neural network model $h_{\phi }$ into $d_{2}$-dimensional instance-level representations $z_{i}=h_{\phi }\left(x_{i}^{\left(1\right)}\right)$ and $v_{i}=h_{\phi }\left(x_{i}^{\left(2\right)}\right)$. The probability of $x_{i}$ being identified as itself (i.e., $x_{i}$) is expressed as:

(2) $$p_{1}\left(i|x_{i}\right)=\frac{\exp\left(z_{i}^{⊤}v_{i}/\tau \right)}{\sum_{i^{\prime }=1}^{N}\exp\left(z_{i}^{⊤}v_{i^{\prime }}/\tau \right)}\;.$$

Here, τ represents the temperature hyperparameter, which controls the concentration level [12]. Contrastive-instance methods construct the loss function via Equation (2) and further learn valuable representations.

Moving to TCC, after generating instance-level representations, it clusters samples and then formulates a loss function centered around the clustering outcomes. This loss function combines the cluster-level and instance-level parts. We first introduce the clustering process of TCC. To allocate *N* samples in $D_{x}$ into *K* clusters, TCC employs learnable clustering parameters $\mu =\left\{\mu_{1},\mu_{2},\cdots ,\mu_{K}\right\}$, where $\mu_{k}\in R^{d_{2}}$, $∥\mu_{k}{∥}_{2}=1$, and ${∥\cdot ∥}_{2}$ refers to the $L_{2}$-norm. Using the dot product to measure similarity between $z_{i}$ and $\mu_{k}$, the membership probability of $x_{i}$ in cluster *k* is calculated as:

(3) $$p_{2}\left(k|x_{i}\right)=\frac{\exp\left(z_{i}^{⊤}\mu_{k}/\tau \right)}{\sum_{k^{\prime }=1}^{K}\exp\left(z_{i}^{⊤}\mu_{k^{\prime }}/\tau \right)}\;.$$

For convenience, we use $\pi_{i}={\left(\pi_{i1},\pi_{i2},\cdots ,\pi_{iK}\right)}^{⊤}\in R^{K}$ to indicate cluster assignment probabilities of $x_{i}$ to each cluster, i.e., $\pi_{ik}=p_{2}\left(k|x_{i}\right)$. Note that $\pi_{ik}$ also reflects the degree of relevance of $x_{i}$ to the *k*-th cluster. With it being the aggregation weight, the representation ${\bar{r}}_{k}$ for the *k*-th cluster can be expressed as follows:

(4) $${\bar{r}}_{k}=r_{k}/{∥r_{k}∥}_{2},r_{k}=\sum_{i=1}^{N}\pi_{ik}\cdot z_{i}\;.$$

Here, $L_{2}$-normalization is adopted for normalized representations benefiting contrastive learning [27].

Analogous to Equation (2), TCC employs representations $v_{i}$ to generate an additional set of cluster-level representations, denoted as ${\hat{r}}_{k}$. Utilizing both ${\bar{r}}_{k}$ and ${\hat{r}}_{k}$, TCC’s cluster-level contrastive objective is formulated as:

(5) $$L_{r}=-\frac{1}{K}\sum_{k=1}^{K}\log\frac{\exp\left({\bar{r}}_{k}^{⊤}{\hat{r}}_{k}/\tau \right)}{\sum_{k^{\prime }=1}^{K}\exp\left({\bar{r}}_{k}^{⊤}{\hat{r}}_{k^{\prime }}/\tau \right)}\;.$$

Minimizing this equation enhances the similarity of representations of the same cluster (${\bar{r}}_{k}$ and ${\hat{r}}_{k}$), while reducing the similarity across different clusters (${\bar{r}}_{k}$ and ${\hat{r}}_{k^{\prime }},k\neq k^{\prime }$), thereby fostering meaningful representations and clustering outcomes.

In addition to the cluster-level contrastive loss function $L_{r}$, TCC also contains the instance-level contrastive loss, the evidence lower bound (ELBO) loss, which is derived from the lower bound of the $\log p_{1}\left(i|x_{i}\right)$. Denote $p_{3}\left(i|x_{i},k\right)$ as the instance identification probability within the context of the *k*-th cluster, with $p_{0}$ denoting the prior following uniform distribution. The relationship between the instance identification probability $\log p_{1}\left(i|x_{i}\right)$ and its lower bound is captured by the following inequality:

(6) $$\log p_{1}\left(i|x_{i}\right)\geq \ell_{elbo}\left(x_{i}\right)≜E_{k∼\pi_{i}}\left[\log p_{3}\left(i|x_{i},k\right)\right]-\mathrm{KL}\left(\pi_{i}∥p_{0}\left(k|x_{i}\right)\right)\;,$$

where KL(·∥·) represents the Kullback-Leibler divergence. The detailed derivation of the inequality can be found in the Appendix A. The right-hand side of this inequality, the ELBO, incorporates the clustering probability $\pi_{i}$ and enhances the clustering performance of TCC. Based on Equation (6), the ELBO loss $L_{elbo}$ for TCC is formulated as:

(7) $$L_{elbo}=-\frac{1}{N}\sum_{i=1}^{N}\ell_{elbo}\left(x_{i}\right)=-\frac{1}{N}\sum_{i=1}^{N}\left[E_{k∼\pi_{i}}\left[\log p_{3}\left(i|x_{i},k\right)\right]-\mathrm{KL}\left(\pi_{i}∥p_{0}\right)\right]\;.$$

By minimizing $L_{elbo}$, TCC maximizes the lower bound of the $\log p_{1}\left(i|x_{i}\right)$, thereby elevating $p_{1}\left(i|x_{i}\right)$. Based on $L_{elbo}$ and $L_{r}$, the loss function for TCC is represented as $L_{TCC}=L_{r}+L_{elbo}$.

### 3.3. Injecting Label Information to TCC

The ELBO loss $L_{elbo}$ is crucial for TCC to generate effective instance-level representations and meaningful clustering results. To align the clustering results more closely with category information, this subsection introduces modifications to $L_{elbo}$.

Note that the KL divergence term $\mathrm{KL}\left(\pi_{i}∥p_{0}\right)$ in Equation (7) involves the clustering prior distribution $p_{0}$, which is simply set as the discrete uniform distribution for lack of meaningful prior information. To enhance the consistency between clustering and classification, it is a feasible way to replace the non-informative prior distribution with a meaningful clustering distribution derived from labels. To implement this replacement strategy, it is necessary to construct a new clustering prior probability distribution related to label information. This motivates us to reflect on the correspondence between classes and clusters.

Utilizing established notations, the total number of classes and clusters is denoted as *C* and *K*, respectively. A one-to-one correspondence between classes and clusters is feasible when C=K, resulting in clustering outcomes that mirror the classification task—whereby each cluster corresponds to a single class. If K<C, a single cluster may encompass multiple categories, diminishing the utility of clustering in identifying similar samples; such configurations are thus excluded from consideration. When K>C, a one-to-one correspondence between clusters and classes cannot be achieved. To extend the concept of correspondence, it is possible to make one class correspond to multiple clusters. This is equivalent to splitting one class into several sub-classes and then associating each sub-class with a cluster. Moreover, a small *K* would pose challenges to TCC training, and *K* usually takes a larger value. Hence, it can be assumed that K>C.

To delineate the one-to-many relationships between classes and clusters, we introduce an alignment matrix $M\in R^{K\times C}$. Ideally, M is expected to realize the transition from the classification probabilities $y_{i}$ to the clustering assignment probabilities $\pi_{i}$, specifically, $\pi_{i}=My_{i}$. For the *k*-th element of the clustering assignment probabilities, the relationship $\pi_{ik}=M_{k,\cdot }y_{i}$ should hold, where $y_{i}={\left(y_{i1},y_{i2},\cdots ,y_{iC}\right)}^{⊤}$, and $M_{k,\cdot }$ represents the *k*-th row of M. For each $\pi_{ik}$, the contribution of $y_{ic}$ to $\pi_{ik}$ is determined by the *c*-th element of $M_{k,\cdot }$, denoted as $M_{k,c}$. Specifically, if cluster *k* is associated with class *c*, then $y_{ic}$ should influence $\pi_{ik}$, signifying that $M_{k,c}>0$; otherwise, $M_{k,c}=0$.

To construct the alignment matrix M, we need to clarify the class correspondences for each cluster. Intuitively, the class correspondence for a cluster should be the majority class label among the samples within that cluster. We refer to the label for the majority of samples as the main class of this cluster. In the context of label-noise classification tasks, there is no access to the true class labels $y_{i}$ and corresponding $y_{i}$ for individual samples. Thus, we resort to using ${\hat{y}}_{i}$ to deduce the class label for each sample, thereby determining the main class for each cluster. Specifically, for the samples within the *k*-th cluster, we estimate the class index for each sample based on ${\mathrm{argmax}}_{c}{\hat{y}}_{ic}$. By aggregating these estimations, we identify the most frequent class, denoted as $m_{k}$, which is considered the main class for the *k*-th cluster. Upon estimating the main class for all clusters, the alignment matrix M is formulated as:

(8) $$\begin{array}{cc} M_{k,c}^{\prime }= & \left\{\begin{array}{cc} 1, & c=m_{k}\;,\\ 0, & c\neq m_{k}\;, \end{array}\right.\\ M_{k,c}= & \left\{\begin{array}{cc} \frac{M_{k,c}^{\prime }}{\sum_{k=1}^{K}M_{k,c}^{\prime }}, & \sum_{k=1}^{K}M_{k,c}^{\prime }\neq 0\;,\\ 0, & \sum_{k=1}^{K}M_{k,c}^{\prime }=0\;. \end{array}\right. \end{array}$$

Here, $M_{k,c}^{\prime }$ is used to indicate the relevance of the *k*-th cluster to the *c*-th class, and $M_{k,c}$ is the result of column-wise normalization of $M_{k,c}^{\prime }$ to ensure that $M{\hat{y}}_{i}$ still satisfy the conditions of the probability distribution.

The KL divergence term in $L_{elbo}$ can be transformed as follows:

(9) $$\mathrm{KL}\left(\pi_{i}∥p_{0}\right)=H\left(\pi_{i}\right)+\ell_{ce}\left(\pi_{i},p_{0}\right)\;,$$

where H(·) denotes the entropy. In the KL divergence term, only the cross-entropy term involves $p_{0}$. With $\mathrm{>M}{\hat{y}}_{i}$ as the new prior, we replace the cross-entropy term with $\ell ce\left(\pi_{i},M{\hat{y}}_{i}\right)$. Simply replacing the prior distribution introduces new pitfalls since ${\hat{y}}_{i}$ may be misled by noise. To mitigate the impact of noisy labels, a confidence threshold γ is introduced to filter out significantly erroneous label information. Specifically, we introduce an indicator function $I(\max_{c}{\hat{y}}_{ic}>\gamma )$. Only ${\hat{y}}_{i}$ satisfying $\max_{c}{\hat{y}}_{ic}>\gamma$ is used to guide clustering. Replace the cross-entropy term in Equation (9) and obtain:

(10) $$\ell_{KL^{\prime }}\left(\pi_{i},{\hat{y}}_{i}\right)=H\left(\pi_{i}\right)+\ell_{ce}\left(\pi_{i},M{\hat{y}}_{i}\right)I\left(\max_{c}{\hat{y}}_{ic}>\gamma \right)+\ell_{ce}\left(\pi_{i},p_{0}\right)I\left(\max_{c}{\hat{y}}_{ic}\leq \gamma \right)\;.$$

Compared to $\mathrm{KL}\left(\pi_{i}∥p_{0}\right)$, Equation (10) introduces category information as a prior into the clustering process, facilitating category-consistent clustering outcomes. It is crucial to note that, during optimization, $M{\hat{y}}_{i}$ is treated as fixed, and only $\pi_{i}$ is updated. The modified ELBO loss is expressed as:

(11) $$L_{elbo}^{\prime }=-\frac{1}{N}\sum_{i=1}^{N}\left[E_{k∼\pi_{i}}\left[\log p_{3}\left(i|x_{i},k\right)\right]-\ell_{KL^{\prime }}\left(\pi_{i},{\hat{y}}_{i}\right)\right]\;.$$

Another key element of $L_{elbo}^{\prime }$ is $E_{k∼\pi_{i}}\left[\log p_{3}\left(i|x_{i},k\right)\right]$, which lies in the construction of $p_{3}\left(i|x_{i},k\right)$. In the original TCC, $p_{3}\left(i|x_{i},k\right)$ is parameterized with a small neural network. However, the introduction of a small neural network added extra parameters, potentially leading to instability in the model training. To enhance the training process’s stability, we utilize the concatenation operation to generate the joint representations, which are subsequently employed to parameterize $p_{3}\left(i|x_{i},k\right)$. Additionally, expectation computation involves the reparameterization trick [39,40]. Specific details can be found in the Appendix B. Finally, the modified TCC loss falls into the following form:

(12) $$L_{TCC}^{\prime }=L_{r}+L_{elbo}^{\prime }\;.$$

### 3.4. Prediction Consistency Regularization Based on Clustering

In the previous section, we adjusted the ELBO loss of TCC to incorporate classification information into the clustering process. In this section, we present a novel regularization term based on clustering results.

The purpose of the regularization term is to eliminate class prediction discrepancies among similar samples. In the clustering process of TCC, by evaluating the similarity between representations and $\mu_{k}$, samples with similar representations are aggregated into the *k*-th cluster. Consequently, from the perspective of representations, samples belonging to the same cluster can be regarded as similar samples. Therefore, the regularization term should ensure that all samples within a cluster have similar class predictions. To achieve this, the most intuitive approach is to constrain differences in class predictions between all pairs of samples. This intuitive approach involves a high computational cost, whereas the prototype-based approach would be more efficient. To develop the prototype-based regularization term, we first generate the prediction center for each cluster and then encourage all class predictions within a cluster close to the corresponding prediction center.

To generate the prediction center, ${\hat{y}}_{i}$ is utilized as the substitute clean label. Note that ${\hat{y}}_{i}$ may contain errors, and not all clustering results have the same reliability. We adopt a weighted averaging approach to overcome potential misleading information. Specifically, for $x_{i}$, we denote its cluster index as $a_{i}=\arg\max_{k}\pi_{ik}$, and the corresponding cluster confidence as $\alpha_{i}=\pi_{ia_{i}}$. Let $M_{k}$ be the set of indices of samples belonging to the *k*-th cluster, then the prediction center for the *k*-th cluster is defined as:

(13) $$\nu_{k}=\frac{1}{\sum_{i\in M_{k}}\alpha_{i}}\sum_{i\in M_{k}}\alpha_{i}{\hat{y}}_{i}\;.$$

Note that $\sum_{c=1}^{C}\nu_{kc}=1$ and $\nu_{kc}\geq 0\left(c=1,2,\cdots ,C\right)$, where $\nu_{k}={\left(\nu_{k1},\nu_{k2},\cdots ,\nu_{kC}\right)}^{⊤}$. This indicates that $\nu_{k}$ remains a probability mass function. Therefore, $\nu_{k}$ can also be understood as an aggregation classification distribution, where clustering confidence $\alpha_{i}$ is the aggregation weight. Based on clustering prediction centers, we construct the regularization term as follows:

(14) $$R=\frac{1}{\sum_{i=1}^{N}\alpha_{i}}\sum_{i=1}^{N}\alpha_{i}\ell_{ce}\left(\nu_{a_{i}},{\hat{y}}_{i}\right)\;.$$

Here, $\ell_{ce}$ represents the cross-entropy function, $a_{i}$ is the clustering assignment for $x_{i}$, and $\nu_{a_{i}}$ is the prediction center of the $a_{i}$-th cluster. Alternative metrics such as inner product [18] could be utilized to quantify the disparity between ${\hat{y}}_{i}$ and its associated prediction center. Equation (14) is also formulated in a weighted averaging manner, which allows samples with higher clustering confidence to have a greater impact and help mitigate the potential impact of clustering errors. Finally, we obtain the following overall loss:

(15) $$L=L_{ce}+L_{TCC}^{\prime }+\lambda R\;,$$

where $L_{ce}$ is the classification loss based on noisy labels, $L_{TCC}^{\prime }$ is the adjusted TCC loss, R is the regularization term, and λ is the regularization strength parameter.

The proposed regularization term relies on the quality of clustering. However, ensuring high-quality clustering during the initial stages of training is often challenging. To prevent the adverse effects of poor clustering results, we introduce a warm-up phase during which the objective function does not include the regularization term. Our training framework is summarized in Algorithm 1.   
**Algorithm 1:** Training Algorithm**Input**: Noisy dataset $\tilde{D}$, total number of training epochs *S*, warm-up epochs $S_{1}$, μ, $f_{\theta }$ and $h_{\phi }$**Output**: Classification network $f_{\theta }$
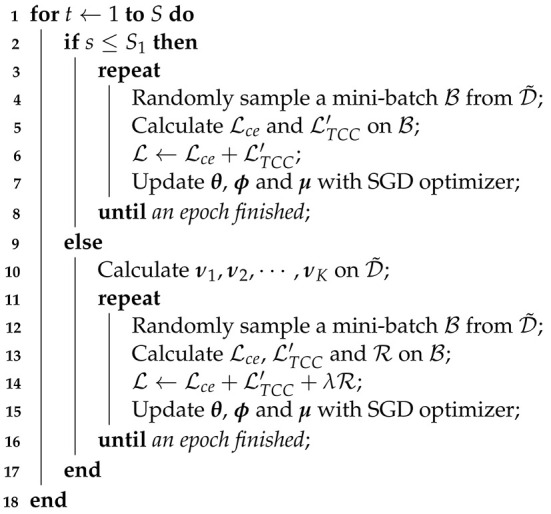


To improve the alignment between clustering and classification while reducing the number of parameters, prior studies frequently shared parts of parameters between $f_{\theta }$ and $h_{\phi }$. This choice is also adopted in this work. More precisely, $h_{\phi }$ is structured as an encoder with the backbone network, while $f_{\theta }$ is the composition of the same backbone network and a classification head.

## 4. Experiment

In this section, we present a series of experiments using synthetic and real-world noisy datasets to confirm the effectiveness of our approach.

### 4.1. Evaluation on Synthetic Noise

We assess the performance of our method on two synthetic noisy datasets, namely CIFAR-10 and CIFAR-100 [41]. Each of these datasets comprises 50,000 training images and 10,000 test images, all with dimensions of 32 × 32 × 3. CIFAR-10 consists of 10 distinct classes, while CIFAR-100 contains 100 classes. We consider two types of synthetic noisy labels, symmetric and asymmetric noise, following the conventions set by previous studies [7,18]. Symmetric noise randomly assigns the labels of the training set to random labels with predefined percentages, a.k.a., noise rates. On the other hand, asymmetric noise considers the class semantic information, and the labels are only changed to similar classes. For CIFAR-10, label flips are performed based on mappings such as “truck → automobile, bird → airplane, deer → horse, cat → dog”. Meanwhile, in CIFAR-100, label flips occur within superclasses in a circular fashion. Our experiments cover various levels of noise. Symmetric noise rates include {0.2,0.4,0.6,0.8}, while asymmetric noise rates include {0.2,0.3,0.4}.

For CIFAR, we use ResNet-34 [42] as the backbone network, and the dimension of output is 128. The classification heads are single-layer networks. We employ the SGD optimizer with a momentum of 0.9 and apply a cosine learning rate decay strategy. The initial learning rate is set at 0.1, and the final learning rate is set at 0.0001. The weight decay is set at $5.0\times 10^{-4}$. We use a batch size of 256 for all experiments. The temperature parameter τ in $L_{TCC}^{\prime }$ is set at 0.2. Before utilizing TPCR, the network is warmed up to 50 epochs. Including warm-up stages, the network is trained for 350 epochs on CIFAR.

The batch size of 256 poses a limitation for clustering and contrastive learning. To address this constraint, we use memory banks [9,12] to help calculate the $L_{TCC}^{\prime }$. For individual representations, the memory bank’s size is 25,600. For cluster-level representations, the size of the memory bank is set as 100×K. Following previous work [10,25], we use random crop, random horizontal flip, and color jitter as augmentation strategies.

For CIFAR, we set the threshold γ as 0.2 in a quantile style. The number of clusters *K* is set at 160 for CIFAR-10 and 200 for CIFAR-100, respectively. For CIFAR-10, λ is set as 1.0 and 0.25 for asymmetric and symmetric noise, respectively. For CIFAR-100, λ is set as 1.0 and 0.5 for asymmetric and symmetric noise, respectively.

We compare our methods to other relevant methods: (1) Standard CE; (2) Forward [43]; (3) GCE [16]; (4) SCE [17]. (5) ELR [18]. (6) GJS [7]. (7) Co-learning [25]. Except for Standard CE, each method employs noise-robust loss functions. Specifically, ELR and GJS are associated with prediction consistency regularization techniques, whereas co-learning utilizes a contrastive learning framework. We re-implement ELR, GJS, and co-learning using publicly available code. To ensure a fair comparison, we present the results of GJS without using RandAug and CutOut data augmentations. All methods employ ResNet-34 [42] as the backbone network. All the experiments are repeated five times with different random seeds, and we report the mean and standard deviation of the best test accuracy. To further demonstrate the efficacy of TPCR, we also report the mean and standard deviation at the last epoch, denoted as TPCR(f).

Table 1 and Table 2 present the test accuracies for CIFAR-10 and CIFAR-100, respectively. As illustrated in Table 1 and Table 2, TPCR exhibits competitive performance when compared to other state-of-the-art (SOTA) methods on CIFAR datasets, thus affirming its effectiveness across various noise scenarios. In particular, for both CIFAR-10 and CIFAR-100, TPCR’s performance is on par with that of ELR and GJS at low noise levels. However, in the presence of high noise levels, TPCR outperforms ELR [18] and GJS [7].

### 4.2. Evaluation on Real-World Noise

We also validated our method on a real-world noisy dataset, Animal-10N [44]. Animal-10N consists of 50,000 training images with complex and confusing appearances, along with 5000 test images, each with a resolution of 64 × 64 × 3 pixels. This dataset comprises 10 classes, with an estimated noise level of approximately 8%. The experiment setting on Animal-10N is the same as experiments on CIFAR-10, except for λ=0.75. We compare our methods to other related methods: (1) Standard CE; (2) Decoupling [20]; (3) Co-teaching [21]; (4) Co-teaching+ [22]; (5) JoCoR [23]; (6) Co-learning [25]. Except for Standard CE, other methods rely on the integration of multiple models or tasks, akin to TPCR. We run TPCR five times and calculate the mean and standard deviation with the best accuracy. We also report the mean and standard deviation of the accuracy at the last epoch (denoted as TPCR(f)). The results of other methods are taken from [25]. All methods use ResNet-34 [42] as the backbone. As shown in Table 3, TPCR surpasses other SOTA methods on ANIMAL-10N, validating the effectiveness of TPCR in real-noise scenarios.

### 4.3. Sensitivity of Hyperparameters

**Figure 1:**
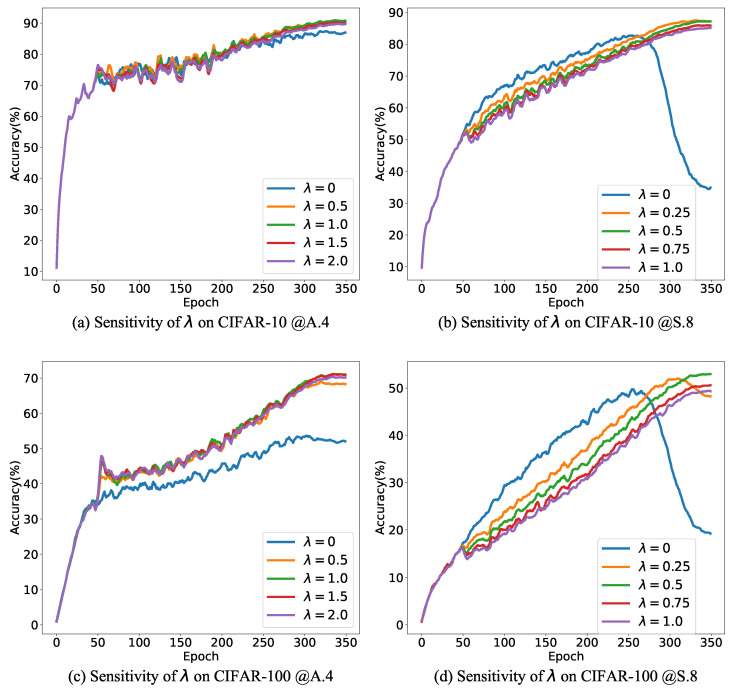
Sensitivity of λ. We show the evolution of test accuracy during training with varying values of λ.

The proposed TPCR involves two crucial hyperparameters: λ and *K*. λ is used to control the strength of the regularization term. λ that is too small may prove insufficient for effectively combating noise, while an excessively large λ could potentially obscure valuable information contained within noisy labels. On the other hand, *K* controls the number of clusters. *K* that is too small can lead to the collapse of the contrastive learning process, which is detrimental to clustering. Moreover, a small *K* may fail to guarantee the quality of clusters. Conversely, an excessively large *K* can result in a limited number of samples within each cluster, diminishing the effectiveness of the regularization term. We conducted an analysis to assess the influence of the regularization strength λ on classification results under 0.4 asymmetric noise (abbreviated as @A.4) and 0.8 symmetric noise (abbreviated as @S.8) settings for both CIFAR-10 and CIFAR-100 datasets. The results, depicted in Figure 1, illustrate the evolution of test accuracy during training with varying values of λ. Notably, the optimal λ value for achieving the highest classification accuracy differs between datasets and noise settings. Generally, both excessively small and excessively large values of λ do not contribute to the best classification accuracy. Furthermore, Figure 1 reveals that different data settings exhibit varying degrees of sensitivity to λ. Specifically, for CIFAR-10 with 0.4 asymmetric noise, λ in the range of {0.5,1.0,1.5} achieves comparable classification outcomes. In contrast, for CIFAR-100 with 0.8 symmetric noise, the preferred value of λ is 0.5. These differences in sensitivity to λ underscore the varying levels of difficulty in mitigating label noise across different scenarios.

**Figure 2:**
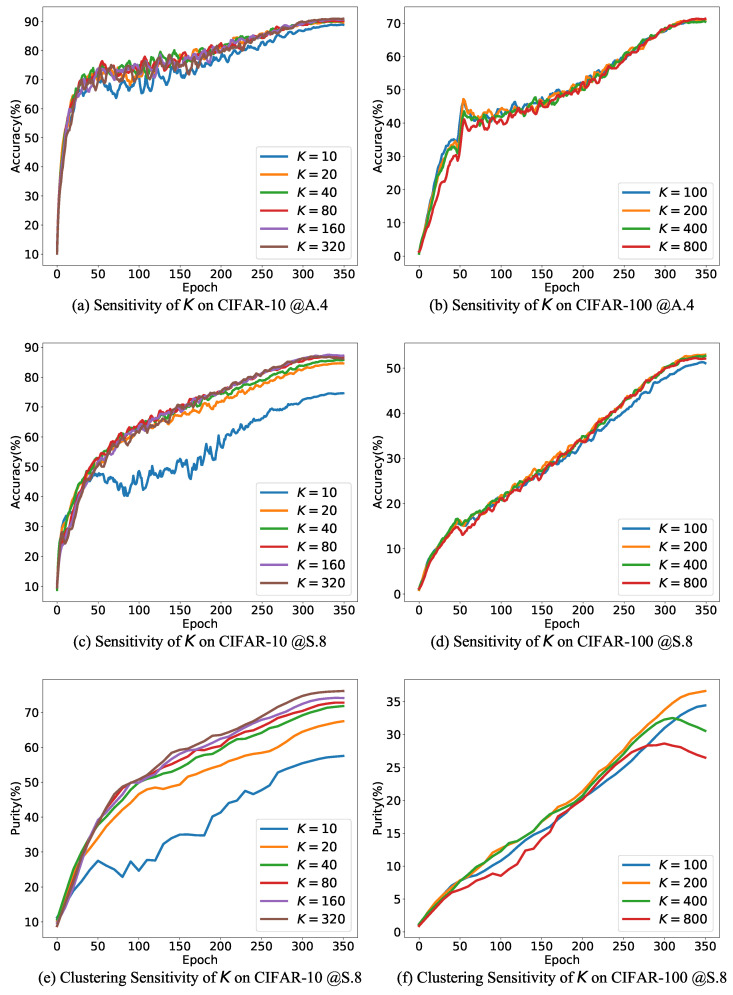
Sensitivity of *K*. (**a**–**d**) show the evolution of test accuracy, while (**e**,**f**) show the evolution of purity on the training set.

Subsequently, we investigated the impact of the number of clusters *K* on our method’s performance in both CIFAR-10 and CIFAR-100 under 0.4 asymmetric and 0.8 symmetric noise settings. The results are presented in Figure 2a–d. As anticipated, excessively small values of *K* prove detrimental to the final classification accuracy. Notably, our method demonstrates resilience to variations in *K*. For CIFAR-10, high classification accuracies can be achieved with K∈{160,320}; for CIFAR-100, high classification accuracies can be obtained by taking K∈{200,400}.

Moreover, *K* emerges as a critical parameter that significantly affects clustering performance. To assess the impact of *K* on clustering performance, we introduce the purity metric, defined as follows:

(16) $$\mathrm{purity}=\frac{\sum_{i=1}^{N}\alpha_{i}I\left(y_{i}=\arg\max_{c}\nu_{a_{i}}\right)}{\sum_{i=1}^{N}\alpha_{i}}.$$

Here, I is the indicator function, $\alpha_{i}$ is the clustering confidence for $x_{i}$, $a_{i}$ is the clustering assignment, and $\nu_{a_{i}}$ is the prediction center of the $a_{i}$-th cluster. The purity metric reflects the degree of consistency between the true labels of individual samples and the prediction center. A purity value of 1 indicates perfect alignment between true labels and cluster predictions, while a value of 0 signifies no consistency between them. The changes in training set purity with varying *K* are depicted in Figure 2e,f. In CIFAR-10 with 0.8 symmetric noise, selecting a small *K*, such as 10, results in lower purity. The potential reason is that a small number of clusters cannot guarantee that all samples within a cluster share the same label, which results in a reduction of purity. Reduced purity, in turn, affects the efficacy of the regularization term, leading to diminished classification accuracy. Higher clustering purity can be obtained when K∈{160,320}. Combining accuracy and purity, for CIFAR-10, 160 and 320 can be used as the recommended values of *K*.

In CIFAR-100 with 0.8 symmetric noise, increasing the number of clusters from 100 to 200 is accompanied by improvements in purity and classification performance. However, further increasing *K* may lead to a decline in purity during later stages of training, indicating a reduction in clustering performance. An intriguing observation is that in CIFAR-100 with 0.8 symmetric noise, a decrease in purity does not necessarily result in an equivalent decrease in prediction accuracy. This may be attributed to the fact that cluster prediction centers employ soft labels. Consequently, even if the maximum probability of the prediction center does not align with the true sample label, as long as the probability associated with the true label is sufficiently high, it can still assist in mitigating label noise. Combining purity and accuracy, the most appropriate value for *K* on CIFAR-100 is 200.

### 4.4. Ablation Study

**Figure 3:**
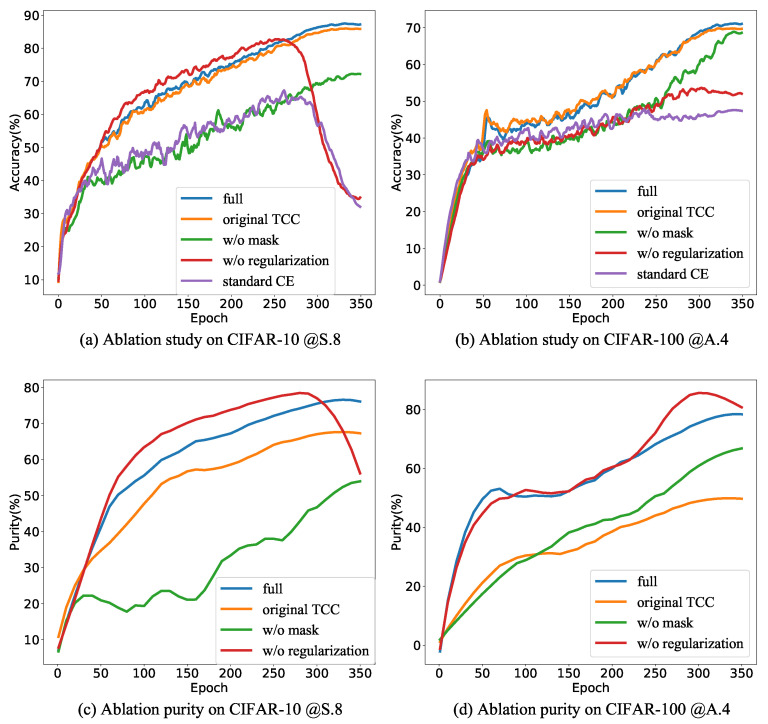
Ablation study. (**a**,**b**) show the evolution of test accuracy, while (**c**,**d**) show the evolution of purity on the training set.

In this section, we conduct an ablation study to validate the effectiveness of the proposed strategies, including the following configurations: (1) Removal of contrastive learning and using only cross-entropy as the regularization term; (2) No adjustment to the evidence lower bound (ELBO) and using the original TCC loss; (3) Direct replacement of the KL divergence term in ELBO without filtering; (4) Removal of the regularization term. Figure 3a,b show the change in test accuracy during training under various configurations, while Figure 3c,d illustrate the evolution of cluster purity during training when the TCC-like loss is included. As shown in the figures, removing any of these components leads to a decrease in the final classification accuracy, confirming the effectiveness of each proposed component.

To elaborate, not adjusting the prior distribution in the ELBO leads to a decrease in cluster purity, consequently causing a decline in classification accuracy. Merely substituting the prior distribution without applying any filtering results in a significant decrease in both cluster purity and classification accuracy. This phenomenon can be attributed to the fact that in the early training stages, when classification predictions are not highly accurate, the prior distribution also exhibits significant bias, which is detrimental to the learning of TCC. Removing the regularization term initially improves classification accuracy during early training stages because the TCC loss provides some resistance to label noise by constraining representations. However, as training progresses, relying solely on the TCC loss cannot completely mitigate label noise, and the model eventually exhibits a decrease in classification accuracy due to overfitting noise.

An intriguing observation is that in Figure 3c,d, removing the regularization term results in an improvement in cluster purity. One possible explanation for this phenomenon is that eliminating the regularization term simplifies the optimization objective, leading to enhanced clustering performance.

### 4.5. Representations Evaluation

**Figure 4:**
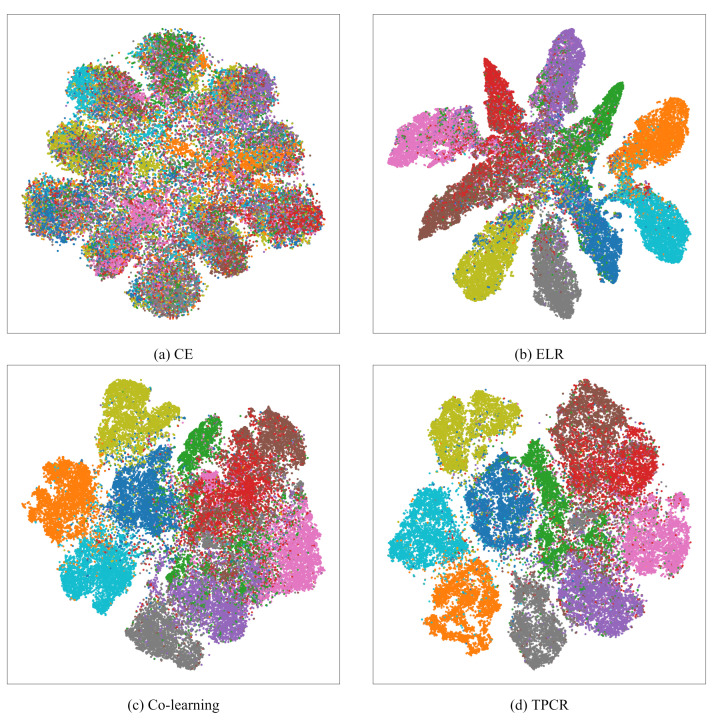
t-SNE Visualization of learned representations on the CIFAR-10 training set with 0.8 symmetric noise. Each color represents a distinct class, and all points are colored according to clean labels.

In this section, we conduct a comparative analysis of the representations generated by TPCR and other methods for a detailed comparison. All methods are trained on CIFAR-10 with 0.8 symmetric noise, and we extract the representations at the output of backbone networks. We then visualize the training set representations in a 2-D space using t-SNE [45]. Figure 4 displays these representations, with distinct colors representing different classes. Compared to the standard cross-entropy (CE) method, all methods, including TPCR, succeed in learning meaningful representations. Notably, TPCR’s representations clearly delineate between categories, unlike ELR and co-learning, which exhibit areas of overlap among different classes. This highlights TPCR’s superior ability to capture distinct and accurate class representations. To further quantify the quality of the representations obtained from different methods, we employ these representations for *k*-nearest neighbor (*k*-NN) classification. Specifically, we derive representations from both the CIFAR-10 test and training set images, subsequently assessing the test set’s classification accuracy using a *k*-NN classifier based on Euclidean distance within the representation space. To ensure a comprehensive comparison of representation quality, we experiment with multiple values for the number of nearest neighbors, applying clean labels, model-predicted labels, and noisy labels to the training set simultaneously. The results, presented in Table 4, reveal that TPCR consistently achieves the highest classification accuracy across all configurations. This performance underscores TPCR’s superiority in generating quality representations compared to other methodologies.

### 4.6. Training Time Analysis

In Table 5, we compare the training times of TPCR with three state-of-the-art methods on CIFAR-10 with 0.8 symmetric noise, using a single Nvidia RTX 3090 GPU. TPCR and co-learning are based on contrastive learning, which takes longer than ELR and GJS. Notably, TPCR’s design obviates the need for computing distances between sample pairs during training, resulting in shorter training times than co-learning.

## 5. Discussion

This paper introduces TPCR as a powerful strategy to handle label noise. TPCR leverages the prediction consistency of multiple instances within the cluster to provide an effective defense mechanism against the adverse effects of noisy labels. To identify similar samples, TPCR has made adjustments to TCC. The modified TCC enables the pretext task of contrastive learning to determine similar samples directly, eliminating the inherent additional computational requirements. Based on the identification of similar samples, we designed the prototypical regularization to guide model training and combat label noise. Experimental results confirm the effectiveness of our method in mitigating noise-induced disruptions. The analysis of experiments demonstrates that the proposed method’s effectiveness stems from the accurate identification of similar samples and the effective design of the regularization term.

While TPCR demonstrates a significant impact, this study has some limitations and potential extensions. Primarily, TPCR’s application has been confined to image data. Nevertheless, the regularization term proposed has the potential for broad applicability across various types of mislabeled data. The challenge lies in adapting twin contrastive clustering (TCC), currently tailored for image data through contrastive learning, to other data modalities. Exploring how to extend TPCR beyond image data presents a promising avenue for future research. Indeed, recent advances in contrastive learning frameworks for non-image data [46,47,48] suggest the feasibility of such an extension. These developments indicate the potential for applying TPCR to more diverse fields, including gene expression and electronic health records, in forthcoming studies.

Furthermore, the design of TPCR’s prediction center and the metric used by the regularization term are relatively straightforward. Constructing more optimal prediction centers and difference metrics represents another research direction that could further enhance noise resilience.

## Figures and Tables

**Table 1:** Test accuracies (%) on CIFAR-10 with different noise settings. All methods use the same backbone, ResNet-34. All results are shown as mean±std.

Method	Sym. Noise Rate				Asy. Noise Rate		
	**0.2**	**0.4**	**0.6**	**0.8**	**0.2**	**0.3**	**0.4**
CE	87.2±0.2	82.3±0.2	75.4±0.5	52.8±0.5	89.0±0.3	86.4±0.4	81.7±0.7
Forward	88.0±0.4	83.3±0.4	75.0±0.7	54.6±0.4	88.3±0.2	86.8±0.4	83.6±0.6
GCE	89.8±0.2	87.1±0.2	82.5±0.2	64.1±1.4	89.3±0.2	85.5±0.7	76.7±0.6
SCE	87.6±0.1	85.3±0.1	80.1±0.1	53.8±0.3	88.2±0.1	85.4±0.1	80.6±0.1
ELR	91.7±0.1	88.4±0.2	86.3±0.6	74.5±0.7	93.1±0.1	91.6±0.3	89.1±0.7
GJS	92.6±0.1	91.1±0.4	87.6±0.4	78.2±0.3	92.1±0.2	90.4±0.6	87.8±0.6
Co-learning	92.2±0.3	91.5±0.2	84.4±0.4	77.6±0.8	91.2±0.3	85.7±0.8	82.5±0.9
TPCR	93.2±0.2	92.7±0.1	89.9±0.3	87.0±0.8	93.3±0.3	92.3±0.3	91.0±0.6
TPCR(f)	93.0±0.2	92.5±0.1	89.5±0.4	86.9±0.8	92.9±0.4	92.2±0.3	90.6±0.7

**Table 2:** Test accuracies (%) on CIFAR-100 with different noise settings. All methods use the same backbone, ResNet-34. All results are shown as mean±std.

Method	Sym. Noise Rate				Asy. Noise Rate		
	**0.2**	**0.4**	**0.6**	**0.8**	**0.2**	**0.3**	**0.4**
Standard CE	60.6±0.4	50.9±0.4	39.5±1.1	21.8±0.8	61.8±0.3	51.2±0.4	44.4±0.2
Forward	39.2±2.6	31.1±1.4	19.1±2.0	9.0±0.6	42.46±2.2	38.1±3.0	34.4±1.9
GCE	66.8±0.4	61.8±0.2	53.2±0.8	29.2±0.7	66.6±0.2	61.5±0.3	47.2±1.2
SCE	60.1±0.2	53.7±0.1	41.5±0.1	15.0±0.1	65.6±0.1	65.1±0.1	63.1±0.1
ELR	73.2±0.2	66.2±0.2	57.1±0.5	30.9±0.6	74.5±0.3	69.3±0.3	66.1±0.3
GJS	73.6±0.2	69.8±0.2	60.6±0.4	35.8±1.1	71.3±0.3	63.2±0.3	54.9±1.4
Co-learning	70.2±0.3	60.4±0.2	52.4±0.4	40.6±0.8	69.5±0.3	60.7±0.3	55.3±0.3
TPCR	74.8±0.3	68.7±0.5	65.1±0.6	53.1±0.8	77.3±0.2	75.4±0.3	71.3±0.6
TPCR(f)	74.5±0.4	68.5±0.5	64.6±0.7	52.9±0.7	76.9±0.4	75.2±0.3	70.0±0.6

**Table 3:** Test accuracies(%) on ANIMAL-10N. All methods use the same model, ResNet-34.

Cross Entropy	Decoupling	Co-Teaching	Co-Teaching+	JoCoR	Co-Learning	TPCR	TPCR(f)
82.68	79.22	82.43	50.66	82.82	82.95	87.62±0.38	87.39±0.24

**Table 4:** Test accuracies (%) of *k*-NN classifier based on representations. *k* is the number of nearest neighbors.

Label	Methods	k=5	k=10	k=50	k=100	k=200	k=500
*y*	ELR	74.51	75.50	75.30	75.18	74.87	74.30
	GJS	78.36	79.30	79.68	79.60	79.68	79.42
	Co-learning	81.42	82.01	81.53	80.88	80.09	79.07
	TPCR	85.27	85.24	85.27	85.08	84.97	84.56
$\hat{y}$	ELR	73.50	73.57	73.55	73.66	73.60	73.65
	GJS	78.44	78.52	78.80	78.82	78.88	78.72
	Co-learning	76.93	77.68	78.25	78.06	77.78	77.27
	TPCR	84.52	84.55	84.76	84.63	84.45	84.18
$\tilde{y}$	ELR	32.97	42.72	69.00	72.75	73.42	73.86
	GJS	30.20	42.10	71.84	76.94	78.82	79.32
	Co-learning	32.57	41.97	71.53	76.57	78.43	78.61
	TPCR	36.74	49.03	79.95	83.49	84.36	84.21

**Table 5:** Comparison of total training time in hours on CIFAR-10 with 0.8 symmetric noise.

ELR	GJS	Co-Learning	TPCR
1.1 h	2.4 h	6.8 h	5.5 h

## Data Availability

We use well-known benchmark datasets [41,44], that have been previously examined in learning with noisy labels.

## Appendix A. Derivation of ELBO

(A1) $$\begin{array}{cc} \log p_{1}\left(i|x_{i}\right) & =\log\sum_{k=1}^{K}p\left(i,k|x_{i}\right),\\ \; & =\log\sum_{k=1}^{K}p_{3}\left(i|x_{i},k\right)p_{0}\left(k|x_{i}\right)\frac{p_{2}\left(k|x_{i}\right)}{p_{2}\left(k|x_{i}\right)},\\ \; & =\log E_{k∼p_{2}\left(k|x_{i}\right)}\left[p_{3}\left(i|x_{i},k\right)\frac{p_{0}\left(k|x_{i}\right)}{p_{2}\left(k|x_{i}\right)}\right],\\ \; & ⩾E_{k∼p_{2}\left(k|x_{i}\right)}\left[\log p_{3}\left(i|x_{i},k\right)\right]+E_{k∼p_{2}\left(k|x_{i}\right)}\left[\log\frac{p_{0}\left(k|x_{i}\right)}{p_{2}\left(k|x_{i}\right)}\right],\\ \; & =E_{k∼\pi_{i}}\left[\log p_{3}\left(i|x_{i},k\right)\right]-\mathrm{KL}\left(\pi_{i}∥p_{0}\left(k|x_{i}\right)\right). \end{array}$$

Here, $p(i,k|x_{i})$ represents the probability of $x_{i}$ being identified as itself and merged into the *k*-th cluster. The inequality stems from Jensen’s inequality, and the final equality arises from replacing $p_{2}\left(k|x_{i}\right)$ with $\pi_{i}$.

## Appendix B. Calculation of the Expectation Term

The expectation term $E_{k∼\pi_{i}}\left[\log p_{3}\left(i|x_{i},k\right)\right]$ is an essential component of the ELBO loss $L_{elbo}$. In this part, we elaborate on the details of its computation. Utilizing conventional Monte Carlo methods to estimate $E_{k∼\pi_{i}}\left[\log p_{3}\left(i|x_{i},k\right)\right]$ can be broken down into two steps: first, get a sample (denoted as $k_{i}$) from $\pi_{i}$, and then calculating $\log p_{3}\left(i|x_{i},k_{i}\right)$. We begin by modeling $p_{3}\left(i|x_{i},k_{i}\right)$. Let the *K*-dimensional one-hot vector corresponding to $k_{i}$ be represented as the bold letter $k_{i}$. We define a joint representation $e_{i}$ using $z_{i}$ and $k_{i}$ as follows:

(A2) $$e_{i}=\frac{{\bar{z}}_{i}⊕k_{i}}{{∥{\bar{z}}_{i}⊕k_{i}∥}_{2}},{\bar{z}}_{i}=z_{i}/∥z_{i}{∥}_{2}$$

Here, ⊕ represents vector concatenation. Following the formulation of $p_{1}\left(i|x_{i}\right)$, utilizing the joint representation $e_{i}$, $p_{3}\left(i|x_{i},k_{i}\right)$ can be parameterized as:

(A3) $$\log p_{3}\left(i|x_{i},k_{i}\right)=\log\frac{\exp\left(e_{i}^{⊤}{\hat{e}}_{i}/\tau \right)}{\sum_{i^{\prime }=1}^{N}\exp\left(e_{i}^{⊤}{\hat{e}}_{i^{\prime }}/\tau \right)}$$

Similarly, ${\hat{e}}_{i}$ is the joint representation constructed based on $v_{i}$.

We aim to differentiate and optimize $E_{k∼\pi_{i}}\left[\log p_{3}\left(i|xi,k\right)\right]$ with respect to the parameters μ,θ,ϕ. However, estimating the expectation term using conventional Monte Carlo methods would result in difficulties in accurately estimating the gradient with respect to $\pi_{i}$. Previous works [39,40] often employ the reparameterization trick to avoid such problems. In this work, we employ the Gumbel-Softmax reparameterization technique [40]. Specifically, to achieve differentiable sampling from $\pi_{i}$, we introduce $c_{i}\in R^{K}$, with each element defined as follows:

(A4) $$c_{ik}=\frac{\exp(\left(\log\pi_{ik}+\epsilon_{ik}\right)/\tau_{2})}{\sum_{k^{\prime }=1}^{K}\exp\left(\left(\log\pi_{ik^{\prime }}+\epsilon_{ik^{\prime }}\right)/\tau_{2}\right)}$$

Here, $\epsilon_{ik}$ is a random variable sampled from the Gumbel distribution Gumbel(0,1), and $\tau_{2}$ is the temperature parameter of the Gumbel-Softmax reparameterization technique, which is fixed at 1.2 in this work. Finally, we replace $k_{i}$ in the joint representation $e_{i}$ in Equation (A2) with $c_{i}$, and substitute it into Equation (A3) to obtain a sample of $\log p_{3}\left(i|x_{i},k\right),k∼\pi_{i}$. Following the VAE [39] and TCC [12], we also use single sampling as the estimate for the expectation term.
